# Sodium and Potassium Consumption in a Semi-Urban Area in Peru: Evaluation of a Population-Based 24-Hour Urine Collection

**DOI:** 10.3390/nu10020245

**Published:** 2018-02-22

**Authors:** Rodrigo M. Carrillo-Larco, Lorena Saavedra-Garcia, J. Jaime Miranda, Katherine A. Sacksteder, Francisco Diez-Canseco, Robert H. Gilman, Antonio Bernabe-Ortiz

**Affiliations:** 1 Center of Excellence in Chronic Diseases (CRONICAS), Universidad Peruana Cayetano Heredia, Lima 18, Peru; rodrigo.carrillo@upch.pe (R.M.C.-L.); lorena.saavedrag@gmail.com (L.S.-G.); jaime.miranda@upch.pe (J.J.M.); fdiezcanseco@upch.pe (F.D.-C.); gilmanbob@gmail.com (R.H.G.); 2 Department of Epidemiology and Biostatistics, School of Public Health, Imperial College London, London W2 1PG, UK; 3 School of Nutrition and Dietetics, Faculty of Health Sciences, Universidad Científica del Sur, Lima 09, Peru; 4 Department of Medicine, School of Medicine, Universidad Peruana Cayetano Heredia, Lima 31, Peru; 5 Department of International Health, Bloomberg School of Public Health, Johns Hopkins University, Baltimore, MD 21205, USA; ksacksteder@gmail.com; 6 Área de Investigación y Desarrollo, Asociación Benéfica PRISMA, Lima 32, Peru; 7 Faculty of Epidemiology and Population Health, London School of Hygiene and Tropical Medicine, London WC1E 7HT, UK

**Keywords:** sodium chloride, sodium, dietary sodium, potassium, dietary potassium, blood pressure, Peru

## Abstract

Despite the negative effects of high sodium and low potassium consumption on cardiovascular health, their consumption has not been quantified in sites undergoing urbanization. We aimed to determine the sodium and potassium consumption in a semi-urban area in Peru with a cross-sectional study. 24-h urine samples were collected. The outcomes were mean consumption of sodium and potassium, as well as adherence to their consumption recommendation: <2 g/day and ≥3.51 g/day, respectively. Bivariate analyses were conducted to identify socio-economic and clinical variables associated with the consumption recommendations of 602 participants, complete urine samples were found in 409: mean age of participants was 45.7 (standard deviation (SD): 16.2) years and 56% were women. The mean sodium and potassium consumption was 4.4 (SD: 2.1) and 2.0 (SD: 1.2) g/day. The sodium and potassium recommendation was met by 7.1% and 13.7% of the study sample; none of the participants met both recommendations. People not adherent to the sodium recommendation had higher diastolic (73.1 mmHg vs. 68.2 mmHg, *p* = 0.015) and systolic (113.1 mmHg vs. 106.3 mmHg, *p* = 0.047) blood pressure than those who comply with the recommendation. Public health actions ought to be implemented in areas undergoing urbanization to improve sodium and potassium consumption at the population level.

## 1. Introduction

The current World Health Organization (WHO) recommendation for sodium intake is 2 g/day [1]. Nonetheless, many populations consume as much as twice the recommendation. The mean sodium intake in 187 countries was estimated at 3.95 g/day [2]. Furthermore, a study carried out in 18 countries found that only 0.2% of the population had a sodium intake <2.3 g/day [3]. A high intake was also reported in Latin America, where a study in Argentina found an average sodium intake of 4.4 g/day [4] in its participants. These figures reflect the large numbers of people who do not comply with the recommendation. However, few studies have reported sodium intake in rural or semi-urban areas, where diverse dietary patterns exist and a different intake of sodium could be expected. For example, in India, slum areas had the highest sodium intake, followed by rural settings and then urban areas; it is noteworthy that differences were as high as 4 g among these settings [5].

High sodium diets increase blood pressure and are associated with increased risk of cardiovascular diseases [6]. Accordingly, lowering sodium intake is associated with a reduction of blood pressure. A mean change in urinary sodium of −1.73 g/day was associated with a mean change of −4.18 mmHg and −2.06 mmHg in systolic and diastolic blood pressure, respectively [7]. Thus, improving sodium intake can reduce hypertension prevalence, which currently reaches 19.7% according to a population-based multi-site study in Peru [8]. This study also reported that, compared to highly-urbanized cities, there was a higher hypertension incidence in semi-urban areas, where the epidemiology of sodium intake has not been studied. Consequently, there is need to fill this gap so that policymakers can develop evidence-based strategies to address unhealthy sodium intake [9].

Another mineral implicated in the regulation of blood pressure is potassium. Potassium increases urinary sodium excretion, thereby diminishing blood pressure. Moreover, potassium induces vascular smooth muscle relaxation and decreases peripheral vascular resistance [10], with a reduction on systolic blood pressure [11]. Evidence demonstrates that the mean potassium intake around the world is 2.1 g/day [12], which is below the WHO recommendation of at least 3.5 g/day [1]. Nonetheless, in comparison to sodium, there are fewer studies that analyze potassium and most have been performed in Asia. The studies conducted in Latin America (in Chile [13] and Brazil [14]) also showed potassium consumption below the recommendation. One study suggested that only 7.9% of the general population evaluated in eighteen countries achieved the recommended potassium consumption [3]. This low adherence to the potassium recommendation is alarming and suggests that analyses addressing the effects of dietary sodium on cardiovascular and other health outcomes should also examine potassium intake [15]. Consequently, the aim of this study was to estimate the mean sodium and potassium consumption using 24-h urine samples in a semi-urban area in Peru.

## 2. Methods

### 2.1. Study Design

This is a cross-sectional analysis of urine samples collected in the context of a randomized stepped wedge trial conducted in Tumbes, Peru. The trial aimed to assess a salt substitute containing potassium chloride to reduce blood pressure at the population level. At the time of this study, only one study village was receiving the intervention. Further details about this implementation study are available elsewhere [16].

### 2.2. Setting

Tumbes is a coastal city at sea level in northern Peru. Overall in the region of Tumbes, according to the Regional Health Office as of 2015, there were 237,686 people (25% aged < 15 years and 4% aged ≥ 65 years), with almost 95% living in urban areas. In addition, 74% of people in Tumbes have at least completed high school [17].

### 2.3. Study Population

The study population for the trial and thus for this analysis, was recruited from the general population (population-based study) living in twenty villages located in semi-urban settings, of which six were randomly selected. In every village, each with 300–600 inhabitants, all households were approached and all eligible subjects were invited to participate. Eligibility criteria included: age ≥ 18 years, being a full-time resident of the area, as well as being able to understand procedures and to give informed consent. Exclusion criteria comprised: having any mental illness and self-reported diagnosis of chronic kidney disease or heart disease [16].

For this study, a random sub-sample was drawn from the original study population, including only one person from each household. Furthermore, for the analysis we included only subjects with a complete 24-h urine sample, defined as: (a) at least 500 mL; and (b) creatinine < 4 mmoL/day for women; or (c) creatinine < 6 mmoL/day for men [18,19].

### 2.4. Variable Collection

Urine samples were collected in a 24-h period and all samples were assessed in the same laboratory facility. Trained health personnel instructed the participants on the correct procedures for collecting the urine sample. Instructions were provided orally and in written format. Because we aimed to study a general population sample in their everyday environment, we anticipated quite a high proportion of incomplete urine samples due to the fact that this request competed with their everyday activities. For example, males mostly work in the field where the lack of toilets facilities could have made it uncomfortable to collect all urine samples throughout the day. Therefore, to optimize data quality, the analysis was conducted with complete urine samples, as defined in the previous section. Because we did not want to interfere with the everyday activities of our participants, no specific procedures were conducted to avoid the risk of incomplete urine samples. We aimed for our results to reflect the sodium and potassium consumption of an average day and not to reflect any influence caused by our study.

Urine samples were used to extract creatinine, sodium and potassium. Creatinine was assessed with the compensated kinetic Jaffe method, while sodium and potassium were assessed with the ion-selective electrode method. Urine samples were retrieved between April and July 2015. In this period, the mean temperature in Tumbes was between 30 °C and 31 °C [20]: therefore, because of the hot weather, there could have been extra sodium losses in sweat.

Blood pressure was assessed after a five-minute resting period. It was measured three times and the mean of the last two measurements was used in the analysis. Trained health staff measured blood pressure using calibrated devices (Omron Healthcare, Inc., Bannockburn, IL, USA). All other variables were collected with standardized paper-based questionnaires. Data collection procedures are detailed elsewhere [16].

### 2.5. Outcome Variables

The outcomes of interest were sodium and potassium consumption. Moreover, adherence to the intake recommendations of sodium and potassium were considered as suggested by the WHO: <2 g/day and ≥3.51 g/day for sodium and potassium, respectively [1]. Furthermore, we also studied the sodium-potassium ratio in three different ways: as a numerical variable, as the proportion of a ratio < 1 and as the proportion of a ratio < 2.

### 2.6. Other Variables

Other variables included in the analyses were sex (women or men); age (18–39, 40–59 and ≥60 years); education (<7, 7–11 and ≥11 years of education); assets index (in tertiles), which is based on household facilities and assets; and village in which the participant lived. Hypertension was defined as: (a) systolic blood pressure (SBP) ≥ 140 mmHg; (b) diastolic blood pressure (DBP) ≥ 90 mmHg; (c) self-reported physician diagnosis; and (d) current use of antihypertensive medication. SBP and DBP were treated as numeric variables and hypertension was dichotomized (yes/no).

### 2.7. Statistical Analysis

All analyses were conducted with STATA 13.0 (StataCorp LLC, College Station, TX, USA). Because there was hot weather during the collection of urine samples and thus there could have been extra sodium losses in sweat, all analyses are presented with laboratory sodium values inflated by 10% as suggested by Johnson and colleagues [5]. Of note, this 10% threshold is a conservative approach, as sweat losses could be higher in hot weather [21]. In addition, we also computed the results with potassium values inflated by 10%. Crude laboratory results (without 10% inflation) for both sodium and potassium as shown in Tables S1 and S2.

To describe numeric variables, we used means and standard deviations, whilst for categorical variables we summarized them as proportions (%). Comparisons between categorical variables were conducted with the Chi-square (Chi-2) test and comparisons between numeric variables were conducted with analysis of variance (ANOVA) and when multiple comparisons were conducted the Bonferroni technique was applied.

Because when urine samples were collected one study village was receiving potassium chloride as part of the original trial, sensitivity analyses were conducted excluding this village.

### 2.8. Ethics

Ethical approval for the implementation study was obtained from the Institutional Review Board of Universidad Peruana Cayetano Heredia (Peru) and the Johns Hopkins University (USA). Written informed consent was obtained from all study participants before any procedure took place. The trial is registered in ClinicalTrials.gov (NCT01960972) [16].

## 3. Results

### 3.1. Characteristics of the Study Population

There were 602 participants who provided a urine sample and 193 (32.1%) did not have a complete urine sample (135 had a sample volume < 500 mL, 74 women had creatinine < 4 mmoL/day and 70 men had creatinine < 6 mmoL/day). Thus, 409 subjects were included in the analysis: 56.0% were women; the mean age was 45.7 (standard deviation (SD): 16.2) years; 37.9% had less than seven years of education, while 16.9% had twelve or more years (Table 1). In sensitivity analysis, Table 1 did not vary considerably, except for the fact that there were no significant differences in age and assets index. The prevalence of hypertension was 17.0% (95% confidence interval (CI): 13.6–21.0%), with a mean DBP and SBP of 72.7 (SD: 10.5) mmHg and 112.6 (SD: 17.6) mmHg, respectively. The 73.9% (95% CI: 62.0–83.1%) of individuals with hypertension reported to be receiving treatment prescribed by a physician.

### 3.2. Sodium and Potassium Intake

The mean 24-h sodium and potassium consumption was 4.4 (SD: 2.1) g/day and 2.2 (SD: 1.3) g/day, respectively. Adherence to the sodium intake recommendation was present in 7.1% (95% CI: 5.0–10.0%) of the studied individuals, whereas this figure for potassium was 13.7% (95% CI: 10.7–17.4%). None of the participants met both recommendations and 91.8% did not meet either recommendation. Mean sodium intake was not different between villages (*p* = 0.71), though there was a difference in potassium intake (*p* < 0.001). The sodium-potassium ratio had a mean of 2.3 (SD: 1.2) g/day. The 4.9% (95% CI: 3.2–7.5%) of the study population had a ratio less than one, though the 48.2% (95% CI: 43.3–53.0%) had a ratio less than two.

In sensitivity analysis, when one village was excluded because it was already receiving the trial intervention, mean sodium and potassium consumption did not change much: 4.3 (SD: 2.0) and 2.0 (SD: 1.2), respectively. Adherence to the sodium recommendation was 8.5% (95% CI: 5.8–12.2%) and adherence to the potassium recommendation was 9.5% (95% CI: 6.6–13.3%). Also in the sensitivity analysis, none of the collected samples showed adherence to both sodium and potassium recommendations and 90.7% did not comply with any recommendation. Furthermore, there were no differences among the remaining villages regarding DBP, SBP, or sodium consumption but there was still a difference regarding potassium (*p* < 0.001). In the sensitivity analysis, the mean of the sodium-potassium ratio was 2.5 (SD: 1.2), the 4.2% (95% CI: 2.5–7.2%) of the study population had a ratio less than one, while the 39.7% (95% CI: 34.4–45.4%) had a ratio less than two.

### 3.3. Factors Associated with Sodium and Potassium Intake

Table 2 shows the characteristics of the participants according to their adherence to the recommended sodium and potassium consumption. Regarding sodium, there were only differences in DBP and SBP, with higher values among those who did not meet the recommendation: 68.2 mmHg vs. 73.1 mmHg (*p* = 0.02) for DBP and 106.3 mmHg vs. 113.1 mmHg (*p* = 0.05) for SBP. With regards to potassium, sex was associated: 62.5% of adherent subjects were men (*p* = 0.003). These results did not change substantially in sensitivity analysis, except for the fact that there was no longer a difference between adherence and non-adherence to the potassium recommendation for a sodium-potassium ratio of less than one.

## 4. Discussion

### 4.1. Main Results

Using 24-h urine samples of a population-based study in a semi-urban area in Peru, our results report a high sodium (4.4 g; SD: 2.1 g) and a low potassium (2.0 g; SD: 1.2 g) consumption. Less than one in ten participants met the sodium consumption recommendation and one in ten met the potassium intake recommendation. No participant met the recommendations for both sodium and potassium consumption.

### 4.2. Comparison with Previous Studies

The mean level of sodium intake found in this study is consistent with other reports in Latin America. Lamelas and colleagues [22] found a mean sodium intake of approximately 4.7 g/day in Argentina, Brazil, Chile and Colombia. The same study showed that sodium intake was higher in rural than in urban areas (4.94 vs. 4.50 g/day) [22]. Thus, these results suggest that there is an unhealthy mean intake of sodium across countries in Latin America, especially in rural areas, suggesting that immediate actions should be taken to improve this dietary pattern in the region.

In our study, the number of people who met the sodium intake recommendation was higher than in other studies in Latin America where only 2.8% of the population met the recommendation [22]; a figure similar to others reported around the world [3]. This could be because our study population lives in semi-urban areas, where most people still consume home-made food and less processed food rich in sodium [23]. The fact that less than one out of ten people met the recommendation is still alarming. As far as potassium is concerned, similar figures to those reported here have been described elsewhere. Mente and colleagues found a mean potassium intake of 2.1 (SD: 0.6) g/day in eighteen countries around the world [3]. Regarding Latin America, O’Donnell and colleagues found that 7.7% of population consumed >3 g of potassium/day [12]. Our results further support the need to reinforce current healthy diet profiles before unhealthy lifestyles become ingrained.

### 4.3. Results Interpretation

The high levels of sodium intake are not surprising because Peruvians use large amounts of salt to prepare meals. A study conducted in Peruvian students found that 8.2 g, from the 10.7 g of salt consumed per day, came from salt added during preparation of meals [24]. Although this study was conducted in an urban setting, results were very similar to ours. Considering that our study population comes from a semi-urban area, the highest proportion of sodium intake would be expected to come from homemade food, because most people in this area eat at home [25]. Moreover, results of a qualitative study revealed that most salt consumption comes from salt added to homemade food [25]. On the other hand, the low potassium intake could be partly explained by the low fruit and vegetable consumption at the national level, where only 10.8% of the population consumes 5 portions per day [26]. Furthermore and particular to our study population, a previous report found that only people with hypertension tried to consume fruits and vegetables on a regular basis but most subjects reported a low consumption of these potassium sources [25].

The low adherence to the recommendations of sodium intake but also of potassium, may be one of the reasons why Tumbes has a higher prevalence of hypertension in comparison to other Peruvian cities [8,27]. But most important, the fact that no participant met the recommendation for both minerals supports the need for urgent actions in public health to improve these diet profiles. Pesantes and colleagues found unhealthy dietary habits in this population but also a dearth of information about the relation between salt intake and hypertension [25]. Therefore, strategies to improve knowledge and awareness of how both sodium and potassium affect our cardiovascular health need to be created. The objective is to decrease sodium intake while increasing potassium consumption concurrently.

We also reported a difference in potassium consumption among the studied villages. This difference could have been explained by the fact that one village was already receiving the intervention (salt with potassium). However, since there were differences among different villages and the sensitivity analysis revealed similar figures, there could be other factors (e.g., fruits/vegetable and consumption) explaining these differences. As part of the implementation of the study, we observed that some villages have faster and better access to more urbanized areas, meaning that some villages could have had easier access to processed foods than others. In addition, it appears that some villages have more convenience stores than others, where people could also access processed foods. In other words, people living in villages far away from urbanized areas, or where the local market does not offer many processed foods, could still rely on fruits, thus having higher potassium intake. Nonetheless, these observations deserve further and systematic exploration, so that potassium sources are clearly identified and enhanced.

Regarding sex as an associated factor to potassium intake, we found that most of the adherent subjects were men. This result is not surprising because men have higher nutritional requirements than women, therefore higher food intake of all kinds [28].

### 4.4. Public Health Implications

Our results are relevant because they fill an important knowledge gap: how much sodium and potassium is consumed at a population level in Peru. A previous study in Peru with university students found a sodium intake that exceeded twice the recommendation [24]; however, this study left unanswered how much the intake at the general population level is. Although the results are not nationally representative, they provide a first approach revealing a worrying scenario.

The results of this study, as well as of other similar initiatives in different sites and populations, could be of interest for policy makers and stakeholders who need this evidence to support the implementation of laws and regulations in favor of healthy diets and to reduce the availability of ultra-processed foods. These kinds of studies are necessary before designing a policy or intervention, because they allow an identification of the main problems and their severity. Our results show that high sodium intake is not the only problem in this region but also low potassium consumption. In this sense, it is important to carry out studies like this that assess both sodium and potassium. Moreover, our results are useful not only to create policies or interventions but to transform the current ones, such as social programs [29] that focus on stunting and nutrient deficiency while forgetting other important profiles in low-income populations, such as high salt, sugar and fat intake.

Our results show that high sodium intake is not the only problem in this region but also low potassium consumption. Different strategies should be implemented in several scenarios to: (i) promote the consumption of home-made foods moderate in salt using natural species and prepared in a short time; and (ii) increase the consumption of fruits and vegetables, especially in a country with great diversity of this type of food. These are challenges that not only correspond to the health sector but also to stakeholders in the education and gastronomy sectors. The former has the responsibility to form new generations with healthy lifestyles; and the latter is part of the Peruvian culinary culture, of which we feel proud and results difficult to give nutritional advice that is not accompanied by good taste and supported by the most influence representatives of the Peruvian gastronomy.

### 4.5. Strengths and Limitations

A major strength of this study was the collection of 24-h urine samples, the gold standard for assessing sodium and potassium intake. In addition, the samples underwent quality control and only complete urine samples were considered in the analysis. Additionally, we considered non-urinary excretion with laboratory sodium values inflated by 10% to provide a result closer to the true values accounting for sodium loss in sweat due to hot weather.

We acknowledge this study has limitations. First, multiple 24-h urine samples are required to get an accurate estimate of a person’s usual sodium and potassium intake and to avoid between-person variation [30]. However, we instead collected a single sample at the population level to estimate population average intake. Second, the study sample was not representative of Tumbes, nor of Peru. Nevertheless, we did not aim to make national estimates but to highlight the sodium and potassium intake in a population undergoing urbanization with high hypertension prevalence. In so doing, we provided valuable information to implement public health actions. Hopefully, these results will create momentum to align other national efforts to improve diets. Third, urine samples were collected when one village was already receiving the intervention, suggesting this could have introduced bias in our results towards higher potassium consumption. However, sensitivity analysis including only villages without the intervention revealed very similar results to those already presented. Fourth, we did not present results of regression models because there were very uninformative point estimates with wide confidence intervals. However, our results are still very important informing of the unhealthy sodium and potassium intake profile in this population. These results may create momentum to conduct larger studies in this topic.

## 5. Conclusions

Sodium consumption is high and potassium consumption is low in the semi-urban population of Tumbes, Peru. The results show higher mean blood pressure in subjects who did not meet the sodium consumption recommendation. Public health actions ought to be implemented to improve these diet profiles at the population level.

## Figures and Tables

**Table 1:** Characteristics of subjects with complete and incomplete 24-h Urine sample ^a^.

Variables	Incomplete Urine Sample (%)	Complete Urine Sample (%)	*p*-Value
Sex	*n* = 193	*n* = 409	0.628
Women	53.9	56.0	
Men	46.1	44.0	
Age	*n* = 193	*n* = 409	0.017
18–39 years	54.4	43.3	
40–59 years	25.4	36.2	
60+ years	20.2	20.5	
Education	*n* = 193	*n* = 409	0.878
<7 years	35.8	37.9	
7–11 years	46.6	45.2	
12+ years	17.6	16.9	
Assets Index	*n* = 192	*n* = 396	0.047
Bottom	34.9	27.8	
Middle	36.5	33.6	
Top	28.7	38.6	
Village	*n* = 193	*n* = 409	0.012
A	11.4	16.6	
B	22.8	19.3	
C	18.7	24.9	
D	8.8	13.2	
E	20.2	12.7	
F	18.1	13.2	
Hypertension	*n* = 192	*n* = 406	0.598
No	81.3	83.0	
Yes	18.8	17.0	
DBP	*n* = 192	*n* = 406	0.388
Mean (SD)	73.5 (11.7)	72.7 (10.5)	
SBP	*n* = 192	*n* = 406	0.232
Mean (SD)	114.5 (19.7)	112.6 (17.6)	
Sodium (Na)	*n* = 193	*n* = 409	<0.001
Mean (SD)	1.7 (1.1)	4.4 (2.1)	
Potassium (K)	*n* = 193	*n* = 409	<0.001
Mean (SD)	0.8 (0.5)	2.2 (1.3)	
Na-K Ratio	*n* = 193	*n* = 409	0.006
<1	10.9	4.9	
≥1	89.1	95.1	
NA-K Ratio	*n* = 193	*n* = 409	0.816
<2	47.2	48.2	
≥2	52.9	51.8	

^a^ Samples were considered complete if urine collection was: (a) at least 500 mL; and (b) creatinine < 4 mmoL/day for women; or (c) creatinine < 6 mmoL/day for men [18]. DBP: diastolic blood pressure; SBP: systolic blood pressure. *p*-values between categorical variables refer to the Chi-square (Chi-2) test, while for numeric variables it refers to the one-way analysis of variance (ANOVA) test. A, B, C, D, E, F: the names of the villages are not reported to secure the confidentiality of the participants. SD: standard deviation.

**Table 2:** Socio-demographic characteristics of the participants according to their adherence to the recommendations of sodium and potassium intake.

Variable	Sodium Adherence		*p*-Value	Potassium Adherence		*p*-Value
	Yes	No		Yes	No	
Sex	*n* = 29	*n* = 380	0.144	*n* = 56	*n* = 353	0.003
Women	69.0	55.0		37.5	58.9	
Men	31.0	45.0		62.5	41.1	
Age	*n* = 29	*n* = 380	0.348	*n* = 56	*n* = 353	0.780
18–39 years	37.9	43.7		39.3	43.9	
40–59 years	31.0	36.6		37.5	36.0	
60+ years	31.0	19.7		23.2	20.1	
Education	*n* = 29	*n* = 380	0.621	*n* = 56	*n* = 353	0.805
<7 years	41.4	37.6		41.1	37.4	
7–11 years	48.3	45.0		44.6	45.3	
12+ years	10.4	17.4		14.3	17.3	
Assets Index	*n* = 26	*n* = 370	0.340	*n* = 54	*n* = 342	0.926
Bottom	19.2	28.4		27.8	27.8	
Middle	46.2	32.7		31.5	33.9	
Top	34.6	38.9		40.7	38.3	
Village	*n* = 29	*n* = 380	0.524	*n* = 56	*n* = 353	<0.001
A	24.1	16.1		8.9	17.9	
B	20.7	19.2		32.1	17.3	
C *	10.3	26.1		48.2	21.3	
D	13.8	13.2		7.1	14.2	
E	13.8	12.6		1.8	14.5	
F	17.2	12.9		1.8	15.0	
Hypertension	*n* = 29	*n* = 377	0.322	*n* = 56	*n* = 350	0.843
No	89.7	82.5		83.9	82.9	
Yes	10.3	17.5		16.1	17.1	
DBP	*n* = 29	*n* = 377	0.015	*n* = 56	*n* = 350	0.149
Mean (SD)	68.2 (9.0)	73.1 (10.5)		74.6 (8.1)	72.4 (10.8)	
SBP	*n* = 29	*n* = 377	0.047	*n* = 56	*n* = 350	0.074
Mean (SD)	106.3 (16.9)	113.1 (17.6)		116.5 (13.7)	111.9 (18.1)	
Na-K Ratio < 1	*n* = 29	*n* = 380	<0.001	*n* = 56	*n* = 353	0.030
Yes	31.0	2.9		10.7	4.0	
No	69.0	97.1		89.3	96.0	
Na-K Ratio < 2	*n* = 29	*n* = 380	<0.001	*n* = 56	*n* = 353	<0.001
Yes	82.8	45.5		87.5	41.9	
No	17.2	54.5		12.5	58.1	

DBP: diastolic blood pressure; SBP: systolic blood pressure; Na: sodium; K: potassium. *p*-values between categorical variables refer to the Chi2 test, while for numeric variables it refers to the one-way ANOVA test. Sodium adherence refers to a 24-h intake of <2.0 g; potassium adherence refers to a 24-h intake of ≥3.510 g. * Village already receiving the intervention by the time of the urine sample collection.

## Supplementary Materials

The following are available online at http://www.mdpi.com/2072-6643/10/2/245/s1, Table S1: Analyses conducted as per laboratory results (not increased by 10%) for sodium and potassium, Table S2: Difference in adherence to the sodium and potassium consumption recommendation as per laboratory results (not increased by 10%).
