# Supplementary material for: Sodium and Potassium Consumption in a Semi-Urban Area in Peru: Evaluation of a Population-Based 24-Hour Urine Collection
Source: Nutrients. 2018 Feb 22;10(2):245. doi: 10.3390/nu10020245 (PMC5852821; doi:10.3390/nu10020245)
Supplement: Supplementary file 1 [file nutrients-10-00245-s001.docx]

**Table S1.** Analyses conducted as per laboratory results (not increased by 10%) for sodium and potassium.

| Mean sodium intake (SD, *n* = 409) | 3.96 (1.86) |
| --- | --- |
| Mean potassium intake (SD, *n* = 409) | 1.98 (1.20) |
| Adherence to sodium intake recommendation (*n* = 409) |  |
| Yes (<2 g/day) | 10.02% |
| No (≥2 g/day) | 89.98% |
| Adherence to potassium intake recommendation (*n* = 409) |  |
| Yes (≥3.510 g/day) | 10.02% |
| No (<3.510 g/day) | 89.98% |
| Were there differences in sodium intake between villages? | No (*p* = 0.714) |
| Were there differences in potassium intake between villages? | Yes (*p* < 0.001) |

**Table S2.** Difference in adherence to the sodium and potassium consumption recommendation as per laboratory results (not increased by 10%).

| Variable | Sodium Adherence | | *p*-value | Potassium Adherence | | *p*-value |
| --- | --- | --- | --- | --- | --- | --- |
|  | **Yes** | No |  | Yes | No |  |
| Sex | *n* = 41 | *n* = 368 | 0.498 | *n* = 41 | *n* = 368 | 0.001 |
| Women | 61.0 | 55.4 |  | 31.7 | 58.7 |  |
| Men | 39.0 | 44.6 |  | 68.3 | 41.3 |  |
| Age | *n* = 41 | *n* = 368 | 0.330 | *n* = 41 | *n* = 368 | 0.770 |
| 18–39 years | 36.6 | 44.0 |  | 39.0 | 43.8 |  |
| 40–59 years | 34.2 | 36.4 |  | 36.6 | 36.1 |  |
| 60+ years | 29.3 | 19.6 |  | 24.4 | 20.1 |  |
| Education | *n* = 41 | *n* = 368 | 0.983 | *n* = 41 | *n* = 368 | 0.701 |
| <7 years | 39.0 | 37.8 |  | 43.9 | 37.2 |  |
| 7–11 years | 43.9 | 45.4 |  | 41.5 | 45.7 |  |
| 12+ years | 17.1 | 16.9 |  | 14.6 | 17.1 |  |
| Assets Index | *n* = 38 | *n* = 358 | 0.623 | *n* = 41 | *n* = 367 | 0.886 |
| Bottom | 21.1 | 28.5 |  | 30.8 | 27.5 |  |
| Middle | 36.8 | 33.2 |  | 30.8 | 33.9 |  |
| Top | 42.1 | 38.3 |  | 38.5 | 38.7 |  |
| Village | *n* = 41 | *n* = 368 | 0.555 | *n* = 41 | *n* = 368 | <0.001 |
| A | 24.4 | 15.8 |  | 9.8 | 17.4 |  |
| B | 22.0 | 19.0 |  | 36.6 | 17.4 |  |
| C | 14.6 | 26.1 |  | 46.3 | 22.6 |  |
| D | 12.2 | 13.3 |  | 7.3 | 13.9 |  |
| E | 14.6 | 12.5 |  | 0.0 | 14.1 |  |
| F | 12.2 | 13.3 |  | 0.0 | 14.7 |  |
| Hypertension | *n* = 41 | *n* = 365 | 0.989 | *n* = 41 | *n* = 365 | 0.388 |
| No | 82.9 | 83.0 |  | 87.8 | 82.5 |  |
| Yes | 17.1 | 17.0 |  | 12.2 | 175 |  |
| Diastolic BP | *n* = 41 | *n* = 365 | 0.081 | *n* = 41 | *n* = 365 | 0.450 |
| Mean (SD) | 70.0 (11.3) | 73.0 (10.4) |  | 73.9 (8.0) | 72.6 (10.7) |  |
| Systolic BP | *n* = 41 | *n* = 365 | 0.282 | *n* = 41 | *n* = 365 | 0.127 |
| Mean (SD) | 109.8 (20.1) | 112.9 (17.4) |  | 116.6 (13.3) | 112.1 (18.0) |  |
| Na-K Ratio <1 | *n* = 41 | *n* = 368 | <0.001 | *n* = 41 | *n* = 368 | 0.002 |
| Yes | 22.0 | 3.0 |  | 14.6 | 3.8 |  |
| No | 78.1 | 97.0 |  | 85.4 | 96.2 |  |
| Na-K Ratio <2 | *n* = 41 | *n* = 368 | <0.001 | *n* = 41 | *n* = 368 | <0.001 |
| Yes | 80.5 | 44.6 |  | 97.6 | 42.7 |  |
| No | 19.5 | 55.4 |  | 2.4 | 57.3 |  |
